# Structure and Function of Hoc—A Novel Environment Sensing Device Encoded by T4 and Other Bacteriophages

**DOI:** 10.3390/v15071517

**Published:** 2023-07-07

**Authors:** Andrei Fokine, Mohammad Zahidul Islam, Qianglin Fang, Zhenguo Chen, Lei Sun, Venigalla B. Rao

**Affiliations:** 1 Department of Biological Sciences, Purdue University, West Lafayette, IN 47907, USA; 2 Bacteriophage Medical Research Center, Department of Biology, The Catholic University of America, Washington, DC 20064, USA; 3 Department of Pathology and Translational Pathology, Louisiana State University Health Science Center, Shreveport, LA 71103, USA; 4 School of Public Health, Sun Yat-sen University, Shenzhen 518107, China; 5 Institutes of Biomedical Sciences, Fudan University, Shanghai 200032, China

**Keywords:** bacteriophage T4, phage head structure, highly immunogenic outer capsid protein Hoc, capsid decoration protein, phage display, antigen display, immunoglobulin-like domains, vaccine development

## Abstract

Bacteriophage T4 is decorated with 155 180 Å-long fibers of the highly antigenic outer capsid protein (Hoc). In this study, we describe a near-atomic structural model of Hoc by combining cryo-electron microscopy and AlphaFold structure predictions. It consists of a conserved C-terminal capsid-binding domain attached to a string of three variable immunoglobulin (Ig)-like domains, an architecture well-preserved in hundreds of Hoc molecules found in phage genomes. Each T4-Hoc fiber attaches randomly to the center of gp23* hexameric capsomers in one of the six possible orientations, though at the vertex-proximal hexamers that deviate from 6-fold symmetry, Hoc binds in two preferred orientations related by 180° rotation. Remarkably, each Hoc fiber binds to all six subunits of the capsomer, though the interactions are greatest with three of the subunits, resulting in the off-centered attachment of the C-domain. Biochemical analyses suggest that the acidic Hoc fiber (pI, ~4–5) allows for the clustering of virions in acidic pH and dispersion in neutral/alkaline pH. Hoc appears to have evolved as a sensing device that allows the phage to navigate its movements through reversible clustering–dispersion transitions so that it reaches its destination, the host bacterium, and persists in various ecological niches such as the human/mammalian gut.

## 1. Introduction

**Figure 1:**
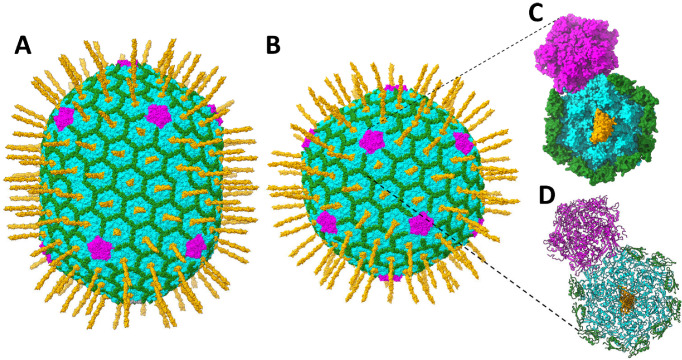
Molecular architecture of the T4 head. (**A**) The native prolate T4 capsid. (**B**) The isometric mutant capsid. The major capsid protein gp23* shell surface is shown in cyan; gp24* vertices are shown in magenta; Soc subunits are shown in green; Hoc fibers are shown in orange. The gp23*, gp24*, and Soc structures were determined in the previous cryo-EM studies of the isometric [7] and prolate capsids [2], whereas the model of the full-length Hoc has been generated for this study. Panels (**C**,**D**) show one pentameric gp24* vertex, one vertex-proximal gp23* hexamer, Soc molecules surrounding gp23*, and Hoc in the center of the gp23* hexamer in surface view (**C**) or in ribbon diagram (**D**).

The bacteriophage T4 virion is composed of a 1200-Å-long and 860-Å-wide prolate capsid or head (Figure 1A) [1,2,3,4] and a 1200-Å-long contractile tail [5]. The capsid contains a ~171 kbp double-stranded genomic DNA, encoding ~300 open reading frames [6]. The capsid shell is made of the major capsid protein, gp23* (“*” represents cleaved mature form), which is organized into a hexagonal lattice characterized by the triangulation numbers T_end_ = 13 for the icosahedral caps and T_mid_ = 20 for the elongated midsection [1,2]. In total, the capsid contains 155 hexameric capsomers of gp23*. Eleven vertices of the capsid are occupied by pentamers of the vertex protein gp24* [7,8], whereas the twelfth vertex is formed by the dodecameric protein gp20, which acts as a portal for the genome to enter the capsid during packaging and exit during infection [9,10,11].

The outer surface of the head is decorated with the highly antigenic outer capsid protein Hoc [12,13,14] and the small outer capsid protein Soc [15] (Figure 1). Both Hoc and Soc are not essential for capsid assembly and attach to the capsid only after its maturational expansion facilitates the creation of the binding sites [2,12,16]. These “decorative” proteins can be bound in vitro to expanded Hoc-minus (Hoc(−)) or Soc-minus ((Soc(−)) mutant heads or phage particles with nanomolar affinity and exquisite specificity [14,15,17]. These features enable the development of T4 into a nanodelivery platform by displaying antigens, targeting ligands, and genome editing molecules for various biomedical applications including vaccines and gene therapeutics [18,19,20,21,22].

The tadpole-shaped Soc subunits attach at the interfaces between adjacent hexameric capsomers and clamp the hexons together (Figure 1) [15]. No Soc molecules are present at the hexamer (gp23*)–pentamer (gp24*) interface or at the hexamer (gp23*)–portal interface [1,2,7,23]. The prolate T4 capsid contains 870 binding sites for Soc. The Soc protein forms an external cage-like structure (Figure 1) through Soc–Soc and Soc–gp23* interactions that stabilize the capsid against extremes in terms of pH, temperature, and other external environmental conditions in the gut [12,24].

The Hoc subunits bind near the centers of gp23* capsomers, one per hexon (Figure 1), and have only a marginal effect on the capsid stability [24]. Therefore, the prolate T4 capsid contains 155 Hoc molecules when the binding sites are fully occupied. The 376-residue Hoc is a monomeric fiber consisting of a string of four domains connected by flexible short linkers, of which the three N-terminal domains have an immunoglobulin (Ig)-like fold [13,14,25]. Though the Hoc protein is nonessential under laboratory conditions, the Ig-like domains probably provide survival advantages in the natural environment [13,26,27,28,29,30,31]. There is evidence to suggest that Hoc binds to bacterial surfaces, which might help the phage to stay attached to the cell while the tail fibers search for the host receptors for infection [13]. This might also allow the virions to travel to different locations that are enriched in the host bacteria [13]. Since *E. coli* and T4 populate the human gut, Hoc may help the phage interact with the cell surface molecules abundant in the gut mucosa [27,28].

Hoc appears to be universally present in the T4 family of phages. Hoc-like fibrous molecules have also been found in T5 and other phage capsids [29,32]. We have previously determined the X-ray structure of the three N-terminal domains of the Hoc protein from the T4-like phage RB49 (Supplementary Figure S1), which shares ~22% sequence identity and ~43% similarity with phage T4 Hoc [13]. The crystal structure showed an almost linear arrangement of the three Ig-like domains. However, the structure of the C-terminal domain, which genetic studies show is responsible for interactions with the capsid [14], could not be determined despite numerous attempts using crystallography, NMR, and cryo-EM approaches. Even the high-resolution cryo-EM reconstructions of the mature isometric and prolate T4 capsids [2,7] did not determine the Hoc structure. The maps showed a featureless dumbbell-shaped density for the Hoc subunits attached to the centers of most gp23* capsomers. Curiously, however, some Hoc features were observed in the hexameric capsomers located near capsid vertices, but the density did not allow for the unambiguous building of the atomic model ab initio [7]. Notably, these hexameric gp23* capsomers show the most significant deviations from the six-fold symmetry due to their interactions with the pentameric vertices made of gp24* [2,7].

In this study, we report that the Hoc density observed in these hexameric capsomers near the capsid vertices is due to two preferred orientations of Hoc that prevail in these capsomers. Furthermore, we present the near-atomic cryo-EM structure of the C-terminal domain of T4 Hoc and delineate its interactions with the gp23* capsid shell. Additionally, we present biochemical data that suggest that Hoc might function as a sensor allowing the phage either to cluster or to disperse depending on the pH of the environment, which might impart survival and fitness advantages to the phage in its natural environment, i.e., the human/mammalian gut.

## 2. Materials and Methods

### 2.1. Hoc Model Building and Refinement

A sequence of the T4 Hoc protein (UniProt P18056) was used to generate a structural model of T4 Hoc using AlphaFold software [33]. The C-terminal domain from the AlphaFold model (residues 281–376) was fitted into the 3.3 Å-resolution cryo-EM reconstruction of the isometric (icosahedral) T4 capsid ([7]; EMDB-8661) at the center of the vertex–proximal gp23* capsomer of the icosahedral asymmetric unit. The cryo-EM map showed two preferred orientations of Hoc. Two chains of the Hoc C-terminal domain, corresponding to the preferred orientations of 1 and 2, were fitted into the cryo-EM density using the Chimera program [34]. The atomic model consisting of the vertex–proximal gp23* hexamer (PDB ID: 5VF3) and two C-terminal domains of Hoc corresponding to the two preferred orientations was refined against the cryo-EM map by adjusting the structure into the density using the Coot program [35] and refinement in real space using the phenix.real_space_refine program [36,37] (Table 1).

Similar preferred orientations of Hoc were detected in the vertex–proximal capsomers of the native prolate T4 capsid [2]. Two chains of the Hoc C-terminal domain, corresponding to the two preferred orientations, were fitted into the 3.4 Å-resolution 5-fold-symmetric reconstruction of the prolate capsid ([2] EMDB-32109) at the center of the gp23* hexamer adjacent to the “top” vertex located on the 5-fold axis. The atomic model consisting of the gp23* hexamer and two C-terminal domains of Hoc was refined against the cryo-EM map using the phenix.real_space_refine program (Table 1).

The two Hoc C-terminal domains were treated as two alternative orientations of the same domain contributing to the cryo-EM density. Therefore, the clashes between the two Hoc chains were ignored during the refinement process. The occupancies of the Hoc C-terminal domains corresponding to the two preferred orientations were refined using phenix.real_space_refine [36,37]. The electrostatic potential was calculated using APBS [38] and mapped to the surface in PyMOL (www.pymol.org). The molecular interface areas were calculated using PISA [39]. Figures were prepared using ChimeraX [40].

### 2.2. In Vitro Assembly of Hoc on T4 Phage Capsid

In vitro assembly (display) of Hoc on T4 phage capsids was performed as described previously [14]. Briefly, T4 Hoc(−) capsids (“(−)“ refers to “minus”, i.e., capsids produced from a Hoc gene deletion mutant and lacking Hoc protein; the same applies to Soc(−)), or T4 Hoc(−) Soc(−) phages, were mixed with the purified recombinant Hoc proteins (T4 Hoc and RB49 Hoc) in a low-bind Eppendorf tube at a 20:1 ratio of Hoc molecules to capsid binding sites in 100 μL of TMG (0.05 M Tris-HCl with 20 mM MgSO_4_) pH 7.4 buffer. Prior to adding them to the reaction mixture, the Hoc protein samples were centrifuged at 34,000× *g* for 45 min to remove any aggregated proteins. The reaction mixtures were incubated at room temperature for 45 min for near-saturation binding of Hoc molecules on T4 capsids, and the samples were re-incubated with various buffer conditions as described below for the sedimentation assays.

### 2.3. Plaque Assay to Determine Clustering vs. Dispersion

In vitro display of recombinant Hoc proteins on T4 Hoc(−) Soc(−) phage was performed as described above in a total reaction volume of 200 μL of Tris-HCl pH 8.0 buffer. After 45 min incubation at room temperature, the samples were centrifuged at 34,000× *g* for 45 min. The unbound Hoc present in the supernatant was discarded, and the bound phage pellet was washed twice with excess buffer to remove any loosely bound Hoc. After resuspension in the same buffer, phages were kept at 4 °C and titrated on *E. coli* B40 on days 0, 1, 3, 7, 10, and 15. The percentage or fold difference of the plaques were calculated by considering the plaque titer at day 0 as 100% (A) or that of T4 Hoc(−) Soc(−) control sample as 1 (B).

### 2.4. Sedimentation Assay to Determine pH Dependent Clustering vs. Dispersion

About 3 × 10^10^ in vitro displayed purified T4 capsids (heads) were incubated in 20 mM Tris-HCl buffer pH 5.6 or pH 8.0 with or without 50 mM NaCl and 25 mM MgCl_2_. After 45 min at room temperature, the capsids were sedimented via centrifugation at 8000× *g* for 45 min. The unbound supernatant was separated from the bound capsid pellet, and the pellet was washed twice with 1 mL of each of the corresponding pH buffers. The final pellets were resuspended in 10 µL of PBS, boiled in the presence of 2× SDS-PAGE sample buffer, and then analyzed by SDS-polyacrylamide gel electrophoresis (PAGE). The gels were stained with Coomassie blue (Bio-Rad, Hercules, CA, USA), and the protein bands were quantified via laser densitometry (PDSI, GE Healthcare, Chicago, IL, USA). The density volumes of gp23* bands were determined for each lane separately, and the % recovery were calculated based on the control lane loaded with 3 × 10^10^ T4 capsids without any centrifugation steps.

## 3. Results

### 3.1. Model of the T4 Hoc Protein

**Figure 2:**
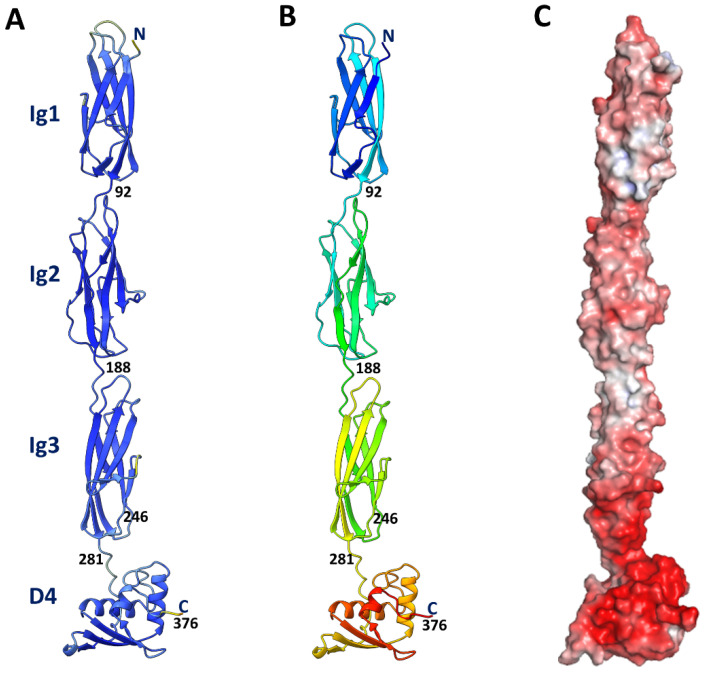
Structural model of the full-length T4 Hoc protein. (**A**) Model of T4 Hoc generated using AlphaFold. The residue colors are based on the pLDDT values [33]. The colors range from blue, corresponding to the pLDDT value of 100 (the highest confidence), to red, corresponding to the pLDDT value of 0 (the lowest confidence). (**B**) The protein chain is rainbow colored from the N-terminus (blue) to the C-terminus (red). (**C**) Molecular surface colored according to electrostatic potential showing the acidic nature of the Hoc protein. The surface color ranges from red, corresponding to a potential of −5 kT/e^−^, to blue, corresponding to a potential of +5 kT/e^−^. The potential was calculated assuming 0 M concentrations for the +1 and −1 ion species.

A structural model was generated for the T4 Hoc protein (376 residues) using AlphaFold software [33] (Figure 2A). Most of the Hoc structure was modeled with high confidence, with ~90% of amino acid residues having pLDDT values greater than 90 [33]. As expected, the model consisted of three N-terminal Ig-like domains and a globular C-terminal domain with α/β topology (Figure 2A,B), and overall, the protein is acidic (Figure 2C).

### 3.2. Vertex–Proximal Capsomers Showed Preferred Orientations of Bound Hoc

**Figure 3:**
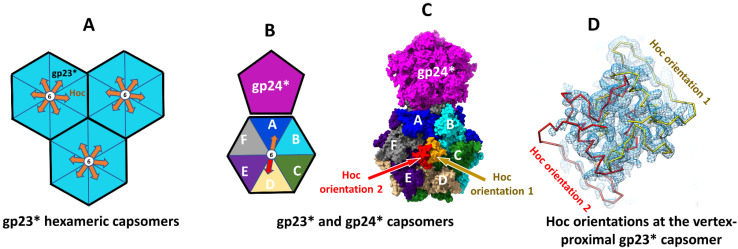
Hoc orientations on gp23* capsomers. (**A**) Schematic representation of three gp23* hexameric capsomers. The six possible orientations of Hoc in the center of each capsomer is depicted by arrows. (**B**) Schematic representation showing the gp24* vertex pentamer as a pentagon and an adjacent gp23* hexamer as a hexagon. The gp23* subunits within the hexamer are shown in different colors. The two preferred orientations of Hoc in the hexamer center are shown by arrows. (**C**) Surface of the gp24* pentamer (magenta) and an adjacent gp23* hexamer. The gp23* subunits are shown in blue, cyan, green, tan, indigo, and gray. Surfaces of Hoc C-terminal domains corresponding to the two preferred orientations are shown in orange and red. (**D**) Backbone traces of the Hoc C-terminal domain in the two preferred orientations (yellow and red) fitted into the cryo-EM density of the isometric T4 capsid reconstruction (EMDB-8661) (blue mesh). The letters A–F in panels (**B**,**C**) represent the major capsid protein (gp23*) subunits of the hexameric capsomer.

Since Hoc monomers bind near the centers of gp23* hexamers, each Hoc monomer can bind in one out of the six possible orientations related by the hexamer axis (Figure 3A). As the prolate T4 capsid contains 155 Hoc binding sites, the number of different possible combinations of Hoc orientations in a capsid would be astronomical, 6^155^, much larger than even the estimated number of all the phages on Earth! Therefore, if Hoc subunits bind in random orientations, each T4 virion would differ from every other virion in its Hoc orientation pattern. Consistent with this, single particle cryo-EM reconstructions, calculated using a large number of phage particles, showed a small volume of dumbbell-shaped Hoc density at the center of most capsomers due to averaging over the six possible orientations [1,14].

However, fitting of the Hoc C-terminal domain (residues 281–376) from the AlphaFold model into the cryo-EM maps at the vertex-proximal gp23* capsomers of the isometric and prolate capsids [2,7] showed only two preferred orientations for the bound Hoc molecules (Figure 3B–D). These two preferred orientations, which gave major contributions to the observed cryo-EM density, are related by 180° rotation around the hexamer axis. The Hoc chains fitted into the density corresponding to the two preferred orientations showed significant overlap, indicating that only one chain can bind to the center of the gp23* hexamer (Figure 3C,D).

### 3.3. Structure of the Hoc C-Terminal Domain Attached to the Vertex–Proximal gp23* Hexamers

The atomic model consisting of the vertex-proximal gp23* hexamer and two C-terminal domains of Hoc corresponding to the preferred orientations was refined against the 3.3 Å-resolution cryo-EM reconstruction of the isometric T4 capsid ([7] EMDB-8661) by adjusting the structure into the density using the Coot program [35], and refinement in the real space was carried out using the phenix.real_space_refine program [36,37]. During refinement, the two Hoc C-terminal domains in the model were treated as two alternative orientations of the same domain contributing to the cryo-EM density. The clashes between the two Hoc chains were ignored.

**Figure 4:**
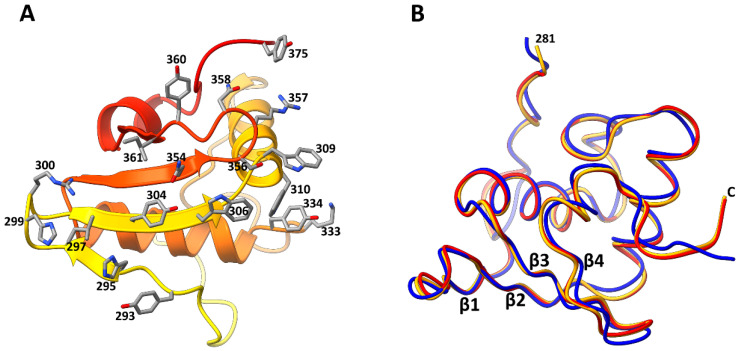
Structure of the T4 Hoc C-terminal domain. (**A**) Bottom view of the Hoc C-terminal domain (residues 281–376) corresponding to the preferred orientation 1. The polypeptide chain color changes from yellow at the residue 281 to red at the C-terminal residue 376. The sidechains of the residues interacting with the gp23* subunits are shown as sticks. (**B**) Superposition of the Hoc C-terminal domain in orientation 1 (orange) with the C-terminal domain in orientation 2 (red) and the C-terminal domain derived from the AlphaFold model (blue).

The resultant refined structure (Figure 4) was in good agreement with the cryo-EM map [7] in terms of the polypeptide fold and positions of large amino acid side chains (Supplementary Figure S2). The refinement of the occupancies for the two Hoc orientations using the Phenix software [36,37] resulted in occupancy values of 0.4 and 0.33 for the preferred orientations 1 and 2, respectively.

The 3.4 Å-resolution five-fold-symmetric reconstruction of the native prolate T4 capsid ([2] EMDB-32109) also shows similar preferred orientations to the Hoc C-terminal domains attached to the vertex-proximal gp23* hexamers. Similar fitting and refinement procedures were performed for the Hoc C-terminal domain attached in the two preferred orientations to the gp23* hexamer adjacent to the “top” pentameric vertex of the prolate capsid located on the five-fold symmetry axis. The occupancy values for the two orientations of Hoc were 0.42 and 0.35, which are close to those in the isometric head.

Since the isometric capsid reconstruction is of a higher resolution and shows stronger density for the Hoc C-terminal domain, here we describe the Hoc-gp23* structure refined against the isometric map.

The C-terminal domain of T4 Hoc contains a four-stranded β-sheet and a three-helix bundle on top of the β-sheet (Figure 2 and Figure 4). The middle β-strands 2 and 3 of the sheet are parallel to each other and are antiparallel to β-strands 1 and 4. The Hoc C-terminal domain has a substantial hydrophobic core in its center made of 14 hydrophobic side chains (Supplementary Figure S3A). The surface contains 16 negatively charged and 11 positively charged residues (pI: 5.1) (Supplementary Figure S3B). The Dali search [41] did not detect any protein domains with significant structural similarities to the Hoc C-terminal domain among the structures deposited into the Protein Data Bank.

### 3.4. Interactions between Hoc C-Terminal Domain and Major Capsid Protein gp23*

**Figure 5:**
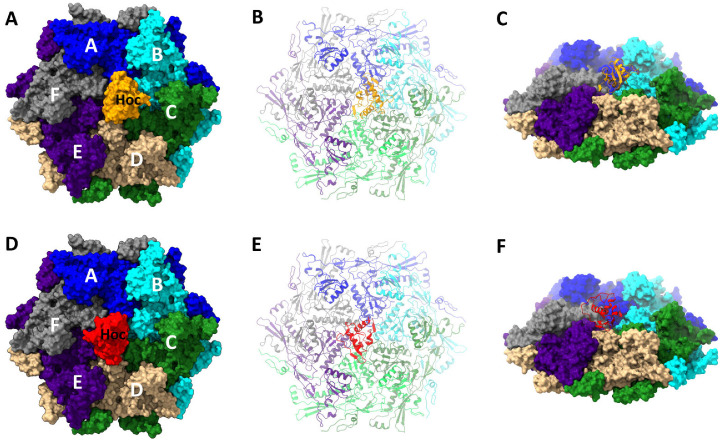
Structure of the T4 Hoc C-terminal domain attached to the center of a vertex–proximal gp23* hexamer. Different gp23* subunits are depicted in different colors. Panels (**A**–**C**) show Hoc C-terminal domain in the preferred orientation 1 (yellow), and panels (**D**–**F**) show the same in the preferred orientation 2 (red). The letters A–F in panels A and D represent the major capsid protein (gp23*) subunits of the hexameric capsomer.

**Figure 6:**
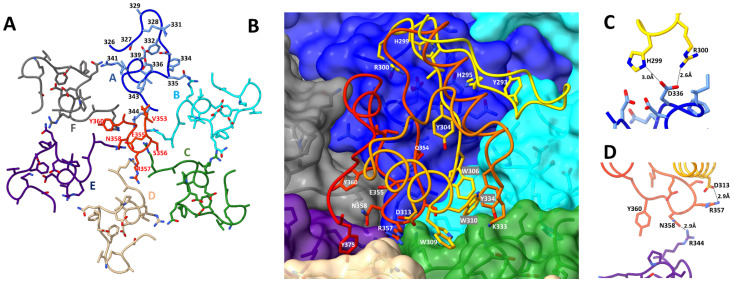
Interactions of Hoc C-terminal domain with the major capsid protein gp23* subunits. (**A**) Regions Gly^326^-Arg^344^ of the six gp23* subunits forming the Hoc binding sites are shown in different colors. The region Val^353^-Tyr^360^ of the Hoc protein in the preferred orientation 1 interacting with gp23* is shown in red. This Hoc region includes the following conserved loop: Glu^355^-Ser^356^-Arg^357^-Asn^358^-Gly^359^. The letters A-F in panel A represent the major capsid protein (gp23*) subunits of the hexameric capsomer. (**B**) The Hoc C-terminal domain in the preferred orientation 1 interacting with the gp23* surface. The polypeptide chain color changes from yellow at the residue 281 to red at the C-terminal residue 376. The side chains of residues involved in Hoc-gp23* interactions are shown as sticks. (**C**,**D**) Close views of some of the Hoc-gp23* interactions.

**Figure 7:**
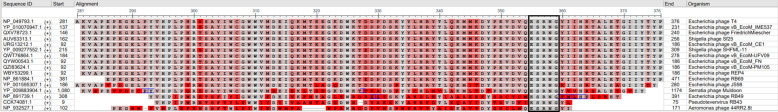
Alignment of Hoc C-terminal domain sequences. Only the nine most homologous sequences found by Blast and the Hoc C-terminal domains of phages RB69, JS98, Muldoon, RB49, RB43, and 44RR2.8t are shown here. For the complete sequence alignment (including 650 sequences), see Supplementary File S1. The residue colors are based on the conservation. The color ranges from gray for the most conserved residues to red for the least conserved residues. The highly conserved Glu^355^-Ser^356^-Arg^357^-Asn^358^-Gly^359^ region is delineated by the black rectangle.

The Hoc C-terminal domain, in both the preferred orientations, interacts with six gp23* subunits with an interface area of ~1200 Å^2^ (Figure 5). The β-sheet region forms a ‘base’, which docks into a groove present in the capsomer (Figure 4, Figure 5 and Figure 6B). There are several bulky side chains on the β-sheet surface (Tyr^293^, His^295^, Tyr^304^, Trp^306^, Gln^354^, and Tyr^360^), which are involved in interactions with the gp23* molecules (Figure 4A and Figure 6B). In addition, the first turn of helix 1 contains two tryptophan residues (Trp^309^ and Trp^310^) interacting with gp23* subunits. Among these residues, Tyr^293^, His^295^, Trp^309^, and Trp^310^ are highly conserved in the sequences of Hoc-like proteins of other phages (Figure 7, Supplementary File S1).

In the preferred orientation 1, which shows the highest occupancy (Figure 5A–C), Hoc interacts most extensively with the gp23* subunit A, which is the closest to the pentameric vertex. The interface area between Hoc and this subunit is ~400 Å^2^, the largest when compared to the other five gp23* subunits. The residues Arg^300^ and His^299^ of the Hoc C-terminal domain, which are located in the loop between β-strands 1 and 2 (Figure 4A and Figure 6B), are involved in charge interactions with the Asp^336^ of the gp23* subunit A (Figure 6C), thus reinforcing the Hoc attachment to the capsid. Remarkably, Arg^300^ is well conserved throughout the Hoc-like proteins of other phages (Figure 7, Supplementary File S1). The Hoc C-terminal domain contains a highly conserved loop region, Glu^355^-Ser^356^-Arg^357^-Asn^358^-Gly^359^, between its β-strands 3 and 4 (Figure 6A,B and Figure 7). Mutations in this region affect the Hoc binding to the capsid [14]. The structure shows that this Hoc region binds to the gp23* capsomer very close to the capsomer’s center, which leads to interactions with all six gp23* subunits (Figure 6A,B). Asn^358^ from this region makes a hydrogen bond with the Arg^344^ of gp23*, and the Arg^357^ residue forms electrostatic interactions with the Asp^313^ of Hoc (Figure 6D). Both Arg^357^ and Asp^313^ are well conserved in the Hoc-like proteins (Figure 7, Supplementary File S1), and the electrostatic interactions between them probably help to maintain the loop in a conformation favorable for attachment to the gp23* capsomers.

The Hoc molecules bound to the hexameric capsomers interact with the region Gly^326^-Arg^344^ of gp23*, which is located near the capsomer axis (Figure 6A). This gp23* region is accessible to solvent in both the unexpanded and expanded T4 capsids [2] and does not show significant conformational changes between the unexpanded and expanded states. However, when the Hoc C-terminal domain structure is placed on the unexpanded gp23* hexamer in such a way that the observed interactions between Hoc and the gp23* subunit A are preserved, the Hoc molecule clashes with the adjacent gp23* subunit B. This explains why Hoc binds to the capsid only after its expansion. During the expansion, the gp23* subunits rotate, and the Gly^326^–Arg^344^ regions of the six subunits assume different relative orientations, which results in the creation of the Hoc binding site in the center of the expanded gp23* capsomers.

### 3.5. Hoc C-Domain Interactions in Alternative Orientations

The Hoc molecule in the alternative preferred orientation 2 is rotated by 180° around the capsomer axis with respect to Hoc in orientation 1 (Figure 5D–F). In this orientation, Hoc shows the most extensive interactions with the gp23* subunit D, which is related to subunit A by the 180° rotation. The interface area between Hoc and subunit D is ~400 Å^2^. Overall, Hoc in orientation 2 forms similar interactions with the gp23* subunits related by the 180° rotation compared to Hoc in orientation 1.

The other four possible Hoc orientations related to orientation 1 by 60°, 120°, 240°, and 300° rotations have minor contributions to the cryo-EM density in the capsomers near the capsid vertices. The Hoc structures in these four orientations cannot be modeled with confidence using the cryo-EM map. However, we generated Hoc in these four orientations by taking the Hoc structure in orientation 1 and rotating it around the capsomer axis by the corresponding angles. The generated Hoc-gp23* models showed unfavorable carbon atom clashes between the Pro^337^ of gp23* exposed on the capsomer surface and the Trp^306^ or Trp^310^ of Hoc. This might explain why these four orientations of Hoc have low occupancies compared with the two preferred orientations.

### 3.6. Structural Models of Phage T4 Full-Length Hoc and of Hoc-like Molecules from Other Bacteriophages

The crystal structure of the RB49 Hoc [13] showed an almost linear arrangement of the three N-terminal Ig-like domains (Supplementary Figure S1). The AlphaFold [33] model of the entire T4 Hoc also showed a linear domain arrangement (Figure 2). Previous biochemical data suggested that the C-terminal domain of T4 Hoc may interact with the first N-terminal domain. Based on these domain interactions, a horseshoe model was proposed [14]. The cryo-EM reconstruction shows the density for the Hoc C-terminal domain in alternative orientations, but does not show any additional density for domain 1 near the C-terminal domain. Therefore, the linear fiber may represent the preferred model.

The Blast search [42] using the T4 Hoc C-terminal domain (residues 281–376) detected 650 homologous protein sequences (Figure 7, Supplementary File S1). Remarkably, most of these Hoc homologs retained the linear fiber architecture containing strings of Ig-like domains, and some phages encode very large Hoc proteins that contain more than a thousand amino acids. For example, the *Serratia* phage Muldoon [43] encodes a Hoc protein containing a total of 1174 residues and 10 N-terminal Ig-like domains followed by the C-terminal domain (Supplementary Figure S4). Since such long sequences are retained in the phage genomes through eons of evolution, it is likely that Hoc provides survival advantages to the virus in its natural environment, such as attaching to surface molecules of bacteria [13] and mammalian cells through the Ig-like domains [27,28,29,30].

Another remarkable phenomenon is the possible use of the C-terminal capsid binding domain to functionalize the capsid surface by displaying other ligands. The phage Muldoon also encodes a second shorter Hoc protein (314 residues) containing two Ig-like domains followed by the C-terminal capsid-binding domain. The enterobacter phage PG7 also encodes two Hoc-like proteins. The shorter protein (233 residues) is composed of an Ig-like domain followed by the C-terminal capsid binding domain. The longer protein (511 residues), however, has the capsid-binding domain on its N-terminus, whereas its C-terminal domain belongs to GDSL-like Lipase/Acylhydrolase family.

### 3.7. Hoc Facilitates Dispersion of Phage T4 Virion Particles

A characteristic feature of T4 and RB49 Hoc proteins is that they are acidic (Supplementary Figure S5) with a pI of ~5, adding substantial acidic character to an already acidic capsid surface (the T4 and RB69 major capsid proteins have a pI of 5.5). The same feature was found in numerous Hoc homologs identified via the Blast search (Supplementary Table S1). This might create a cloud of negative charges surrounding the virion. Therefore, the electrostatic repulsion imparted to the Hoc-displayed viral particles might keep the particles separate and help prevent aggregation while enhancing viral dispersion, which is essential for efficient infection.

**Figure 8:**
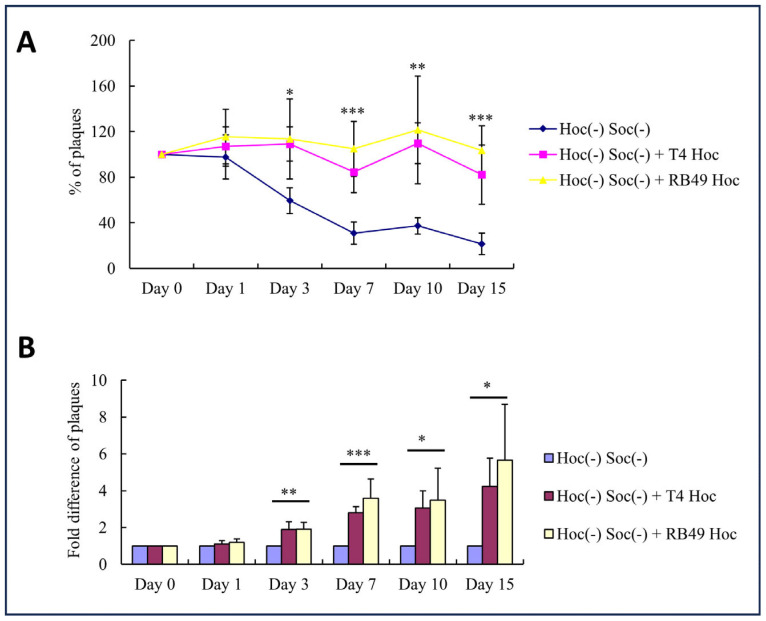
Clustering behavior of T4 phage with and without Hoc. Plaque assays were performed after incubation of Hoc(−) Soc(−) phage with or without the recombinant T4-Hoc or RB49-Hoc at 4 °C. Percentages and fold differences of plaques were calculated by taking the plaque titer of control Hoc(−) Soc(−) phage as 100% (**A**), or 1 (**B**). Error bars were determined from quadruplicate assays. * *p* < 0.05, ** *p* < 0.01, and *** *p* < 0.001, one-way ANOVA test.

To test the potential impact of Hoc on particle dispersion, plaque-forming titers of phage suspensions were determined over a period of two weeks. If aggregation occurred, there would have been a loss of plaque-forming titer. Our results show that a gradual loss of plaque titer occurred over time in Hoc(−) Soc(−) phage particles compared to the same decorated with the recombinant Hoc protein, either the T4 Hoc or the RB49 Hoc (Figure 8). On day 15, the Hoc(−) Soc(−) phage titer was reduced to ~20–25% when compared to the titer on day 1, whereas similar titers (80–100%) were observed with the T4 or RB49 Hoc(+) phages. Since the Hoc proteins do not affect the capsid stability, the reduction in plaque titer in Hoc(−) phage may be due to aggregation of the virions in the absence of the acidic Hoc molecules.

Furthermore, we observed that, when supplemented with the recombinant Hoc, even the wild-type (WT) phage produced 1.8 times more plaques on day 15 compared with the unsupplemented WT phage (Supplementary Figure S6). Similarly, the addition of recombinant RB49 Hoc to the WT T4 phage resulted in 2.2 times more plaques on day 15 compared with the unsupplemented WT virus. These results indicate that some of the Hoc binding sites are not occupied in the WT virions and that saturation with the externally added Hoc protein to full occupancy (155 per capsid) further increases the electrostatic repulsion and leads to better dispersion of the virions.

### 3.8. Hoc Might Function as an Environmental Sensor

Could the long, acidic, antenna-like Hoc fibers serve as sensors of the gut environment, particularly the pH? One of the main environmental niches in which the T4 phage resides is the human/mammalian gut. The pH of the gut shifts from highly acidic in the stomach (~pH 1) to neutral or slightly alkaline in the small intestine (~pH 7–8) [44]. The *E. coli* host bacteria are present largely in the small intestine. Therefore, it is plausible that Hoc might help cluster (aggregate) the virions in acidic pH, as it is closer to its pI. Clustering would protect the integrity of the phage in the acidic environment and minimize the nonspecific attachment of virions to surfaces. On the other hand, in the neutral or slightly alkaline pH environment of the small intestine that is enriched with the host *E. coli*, the ionization of acidic groups and electrostatic repulsion would lead to unclustering and virion dispersal for efficient infection.

**Figure 9:**
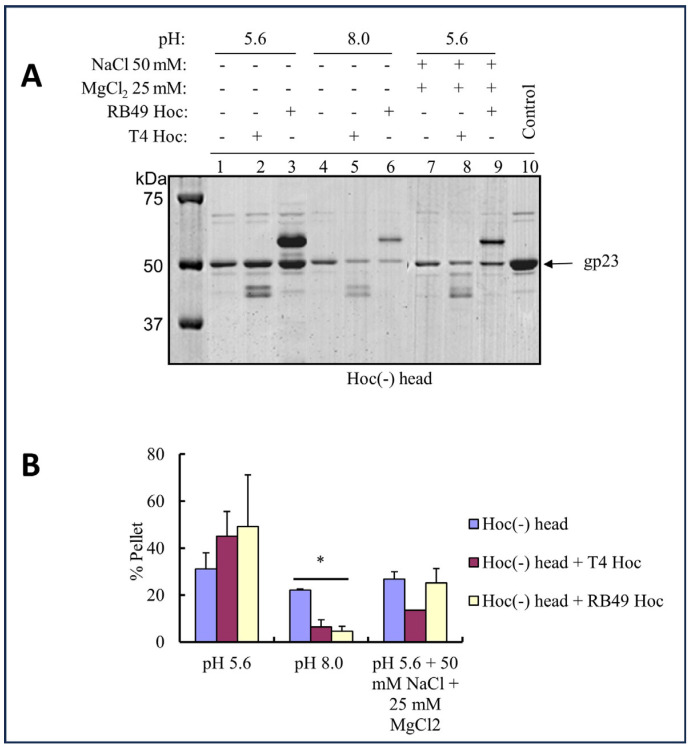
The dispersion of T4 heads (capsids) is dependent on Hoc. (**A**) Purified T4 capsids were incubated in pH 5.6 (lane 1 to 3) or pH 8 (lane 4 to 6) buffers either with or without the recombinant T4 or RB49 Hoc and in the presence or absence of salts. The samples were sedimented at 8000× *g* for 45 min, and SDS–PAGE analysis of the pellets was performed. (**B**) The volume of gp23* band from each lane was determined by ImageDoc, and bar graphs were plotted using the pixel values. The volume of gp23* of Hoc(−) head control was considered as 100%. Error bars were determined from duplicate assays. * *p* < 0.05, one-way ANOVA test.

To test this hypothesis, we evaluated the aggregation behavior of Hoc(−) and Hoc(+) phage T4 capsids in acidic (pH 5.6) and alkaline (pH 8) buffers. Hoc(−) capsids and Hoc(+) capsids saturated with either T4 Hoc or RB49 Hoc were centrifuged at 8000× *g* for 45 min to sediment capsid aggregates while ensuring the retention of the dispersed particles in the supernatant. Capsids were used for this experiment to avoid interference from the tails. The results (Figure 9) show that, at pH 5.6, up to 45–50% of Hoc(+) capsids were sedimented, whereas at pH 8, only 5–7% of the capsids were sedimented. On the other hand, Hoc(−) capsids (which are also negatively charged due to the acidic gp23*) showed fewer differences in sedimentation behavior (~30% at pH 5.6 and ~22% at pH 8).

The sedimentation of a significant fraction of the Hoc(+) T4 capsids at pH 5.6 compared to pH 8.0 can be explained by the reduced charges of Hoc molecules near its acidic pI, as a result of which the electrostatic repulsion was reduced. Consequently, Hoc increases capsid aggregation at an acidic pH close to its pI but increases dispersal at pH 8, which is far from the pI. Furthermore, salts reduced aggregation and increased dispersal. In the presence of 50 mM NaCl and 25 mM MgCl_2_ at pH 5.6, the percentage of Hoc(+) sedimented capsids in the pellet was reduced by ~50% (Figure 9), probably because, by promoting polar interactions between the proteins on the capsid surface and the aqueous solvent, the salts partially compensated for the loss of charges at a pH near the pI of Hoc.

The above-mentioned two sets of data (Figure 8 and Figure 9) suggest that the long acidic Hoc fibers might help phage to cluster or disperse depending on the environmental conditions.

## 4. Discussion

In this study, we described the structures of the C-terminal capsid binding domain of phage T4 Hoc and its complex with the gp23* hexameric capsomers. By combining these with our previous studies and AlphaFold modeling, a complete structural model for the full-length four-domain Hoc can be generated. The result is a remarkable fiber with a conserved C-terminal capsid binding domain attached to a string of variable Ig-like domains that vary in terms of the sequence, size, and flexibility of inter-domain linkers. This overall Hoc architecture seems to be preserved among hundreds of Hoc genes found in phage genomes.

The results from our structural analyses further suggest that the Hoc fibers attach randomly to the centers of the gp23* hexamers in one out of the six possible orientations related by the rotations around the six-fold axis. However, at the gp23* hexamers proximal to the capsid vertices, which deviate from the six-fold symmetry, Hoc binds in two preferred orientations, which are related by 180° rotation, in part because the other orientations involve clashes and, most likely, less stable interactions.

Remarkably, each Hoc subunit attached to the capsid interacts with all the six subunits of the gp23* hexamer. This indicates evolutionary optimization to firmly fix one end of the fiber, the C-domain, to the capsid. However, the position of the C-domain is off-center because three of the subunits form multiple interactions, whereas the other three have limited interactions. Curiously, the ~1200 Å^2^ interaction interface contains eight bulky aromatic residues of Hoc, including three tryptophans, which might provide a strong hydrophobic interacting surface.

By randomizing the Hoc orientations on the capsid, T4 virions would display essentially all possible orientations of Hoc fibers distributed on the capsid surface. Furthermore, since the features of the Ig-like domains are variable, it would provide a large evolutionary space to functionalize the capsid surface. This is a striking observation, as we examined hundreds of Hoc fiber genes from various phages, each different in its sequence from another but all having similar overall architecture. Even the closely related T4-family phages show great variability in the Ig-like domains. Furthermore, one of the largest Hoc-like molecules, which belongs to the *Serratia* myophage Muldoon [43], consists of 1174 residues and has 10 Ig-like domains. These features implicate the evolution of Hoc genes at a significant genome cost, probably because Hoc provides considerable survival advantages in the ecological niches the phages reside in.

Biochemical analyses suggest that Hoc helps the phage to respond to the environment, in particular to pH, either to cluster or to disperse. The acidic nature of T4 Hoc and its numerous homologs (pI, ~4–5; Supplementary Table S1) is a telling characteristic. The pH might be particularly relevant in the mammalian gut, which is enriched in bacterial microbiome and where shifts in pH are massive and an integral part of gut anatomy. In an acidic environment (close to Hoc’s pI), there are fewer charges on the acidic capsid surface as well as less electrostatic repulsion between particles that favor the clustering of virions. At neutral or alkaline pH, due to the ionization of acidic groups, the repulsion of negatively charged particles allows for dispersion. Our data show that plaque titers are reduced by as much as 75–80% in the absence of the Hoc, presumably due to clustering. In line with our observations, Szermer-Olearnik et al. [45] showed the salt-dependent reversible aggregation and disaggregation of phage T4 and corresponding changes in plaque titers. Thus, it is reasonable to speculate that T4 phages sequester into ordered clusters through Hoc–Hoc interactions that might confer protection in the damaging acidic environment of the gut and reverse dispersion in the neutral environment of the small intestine for efficient infection of the resident host bacteria.

The picture emerging from the above analyses is that Hoc is a device evolved by phages to sense the environment and provide survival advantages in various ecological niches. This might be manifested in several forms. First, up to 155 negatively charged Hoc fibers extending out to a distance of ~180 Å from the capsid provide a significant range of interactions between the virion and the environment. Inside the *E. coli* cell where virions assemble, it is essential that the assembled capsids do not aggregate. The acidic Hoc molecules bound to the capsid after expansion help the matured capsids disperse in the pH-neutral intracellular environment. Dispersion, rather than clustering, allows for the efficient operation of downstream processes such as genome packaging and tail docking, which are essential for virion assembly.

Second, when the virions are released into the external environment, Hoc might help the virions to reversibly attach to bacterial cell surfaces. Which bacterial surfaces it would attach could be varied through evolution of appropriate Ig-like domains. The phage can then use the bacterium as a vehicle to travel to different locations. Since bacteria move towards nutrient-rich locations where other potential hosts would also congregate, “piggybacking” on the bacteria may be beneficial for phage propagation.

Third, Hoc might help the phage stay attached to the *E. coli* bacterium when the conditions are unfavorable for infection. We previously reported that both T4 Hoc and RB49 Hoc, in particular the Ig-like domains 2 and 3, attach to *E. coli* surfaces [13]. Furthermore, it is well known that the T4 long tail fibers (LTFs) assume the “up” conformation in unfavorable conditions, e.g., under low temperatures, acidic pH, or low ionic strength [46]. In this “up” configuration, LTFs extend along the tail, interacting with the whiskers and the tail sheath, meaning that they are less likely to bind to LPS and/or OmpC receptors [47]. When favorable conditions return, the Hoc binding is reversed, causing virion detachment and the LTFs switch to “down” configuration, allowing for efficient infection. Thus, Hoc, whiskers, and LTFs might coevolve as a sensory machine to respond to the environment for better infectivity.

Fourth, as mentioned above, Hoc might help phage navigate its movements in the human gut by clustering in the unfavorable acidic environment of the stomach and dispersion in the favorable neutral/alkaline environment of the small intestine (where *E. coli* resides). Barr et al. [28] reported that T4 Hoc binds to the fucosylated glycan moieties of mucin glycoproteins present in mucosal secretions. An Asp^246^ to Asn mutation in domain 3 was selected when T4 phage was serially grown on a gut-on-a-chip ex vivo model, apparently providing a survival advantage in this environment [27].

Fifth, through reversible interactions with mucin and with other surface proteins of the gut mucosa and the microbiome, T4 phages might persist in the mucosal environment even when the host population is depleted and be able to be infective when the host population returns [27,28,31]. It has been well established that surface-exposed Ig-like domains confer adhesive properties in many biological systems through the recognition of cell surface molecules [48,49,50,51].

In conclusion, our T4 Hoc structure–function studies reveal that Hoc is a molecular sensor that T4 and other bacteriophages are equipped with on the virion surface. Through weak reversible interactions with bacteria and gut mucosal surfaces and clustering and dispersal transitions, T4 might be able to sense the environment and navigate its movements for better survival and infectivity. Therefore, a dynamic evolutionary relationship exists among phages, microbiome, and mucosa to evolve diverse Hoc molecules. Though Hoc is not an essential element of the structure, it nevertheless appears to be essential for the successful navigation of the virion payload in the vast external environment to reach its host destination. Therefore, it is not surprising that Hoc-like molecules are commonly found in numerous phage genomes. Other phages display Ig-like domains on the capsid surface as direct insertions into the major capsid protein [29], presumably carrying out a similar function. That viruses, the smallest “organisms” on Earth, are endowed with such devices, just as bacteria and higher organisms are, should open new avenues for mechanistic investigations and biotechnology applications. Often though, these components are brushed aside as “nonessential” based on a limited definition of “essentiality” under artificial and narrowly defined laboratory conditions. However, in the natural world, these devices may be as essential or even more essential for virus survival than some of the so-called essential components.

## Figures and Tables

**Table 1:** Structure refinement statistics.

	Hoc-gp23 in Isometric Capsid	Hoc-gp23 in Prolate Capsid
R.m.s. deviations		
Bond lengths (Å)	0.003	0.007
Bond angles (°)	0.62	0.69
Dihedral angles (°)	4.84	13.50
Ramachandran plot		
Favored (%)	91.1	90.3
Allowed (%)	8.5	9.1
Disallowed (%)	0.4	0.6
Rotamers outliers (%)	0.67	5.53
Clashscore	13.86	12.84
CC_mask_	0.85	0.84

## Data Availability

The atomic coordinates of the Hoc C-terminal domain in two preferred orientations bound to vertex-proximal gp23* capsomers have been deposited in the Protein Data Bank (PDB) with accession codes 8T1X for the isometric capsid and 8T9R for the prolate capsid.

## Supplementary Materials

The following supporting information can be downloaded at: https://www.mdpi.com/article/10.3390/v15071517/s1, Figure S1. Crystal structure of the three Ig-like N-terminal domains of RB49 Hoc (PDB ID: 3SHS). Figure S2. The atomic structure of the T4 Hoc C-terminal domain in the preferred orientation 1 fitted into the cryo-EM density of the isometric T4 capsid (EMDB-8661). Figure S3. Top view of the T4 Hoc C-terminal domain. The polypeptide ribbon color changes from yellow at the N-terminal residue 281 to red at the C-terminal residue 376. (A) Side chains of the residues forming the hydrophobic core are shown as sticks. (B) Side chains of the charged residues (Arg, Lys, Asp, and Glu) are shown as sticks. Figure S4. Model of the Hoc protein from phage Muldoon created using AlphaFold. The protein chain is rainbow colored from the N-terminus (blue) to the C-terminus (red). Figure S5. Molecular surface of the T4 Hoc model showing changes in the electrostatic potential depending on the salt concentration. The color ranges from red, corresponding to a potential of −5 kT/e^−^, to blue, corresponding to a potential of +5 kT/e^−^. For the left panel the electrostatic potential was calculated using 150 mM concentrations for the +1 and −1 ion species. For the right panel the potential was calculated using 0 M ion concentrations. Table S1. Isoelectric points (pI) of Hoc proteins. Figure S6. Clustering behavior of WT phage by supplementation with additional Hoc. Plaque assays were performed after the incubation of WT phage with or without the recombinant T4-Hoc or RB49-Hoc at 4 °C. Percentages and fold differences of plaques were calculated by taking the plaque titer of control WT phage as 100% (A) or 1 (B). File S1. Alignment of Hoc C-terminal domain sequences. The residue colors are based on the conservation. The color ranges from gray for the most conserved residues to red for the least conserved residues.
