# Supplementary material for: Structure and Function of Hoc—A Novel Environment Sensing Device Encoded by T4 and Other Bacteriophages
Source: Viruses. 2023 Jul 7;15(7):1517. doi: 10.3390/v15071517 (PMC10385173; doi:10.3390/v15071517)
Supplement: Supplementary file 1 [file viruses-15-01517-s001.zip › Supplementary_Table_S1.pdf]

Supplementary Table S1. Isoelectric points (pI) of Hoc proteins

Hoc protein	pI value
T4 Hoc	4.74
44rr2Hoc	4.23
RB49 Hoc	4.57
RB69 Hoc	4.72
RB43 Hoc	5.16
JS98 Hoc	4.45
Muldoon Hoc	4.70
