# Supplementary figures and images for: Structure and Function of Hoc—A Novel Environment Sensing Device Encoded by T4 and Other Bacteriophages
Source: Viruses. 2023 Jul 7;15(7):1517. doi: 10.3390/v15071517 (PMC10385173; doi:10.3390/v15071517)

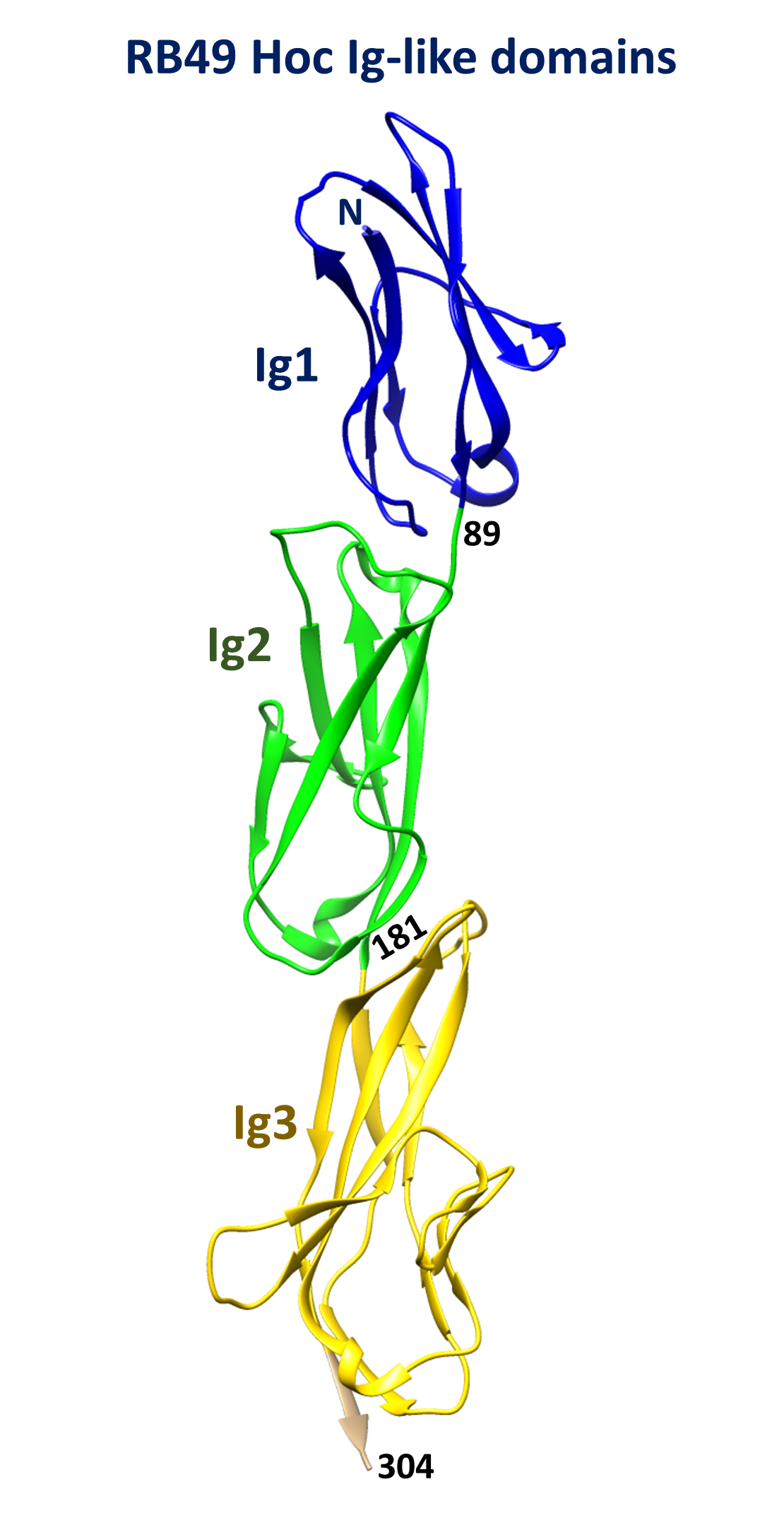

Supplement: Supplementary file 1 [file viruses-15-01517-s001.zip › Supplementary_Figure_S1.png]

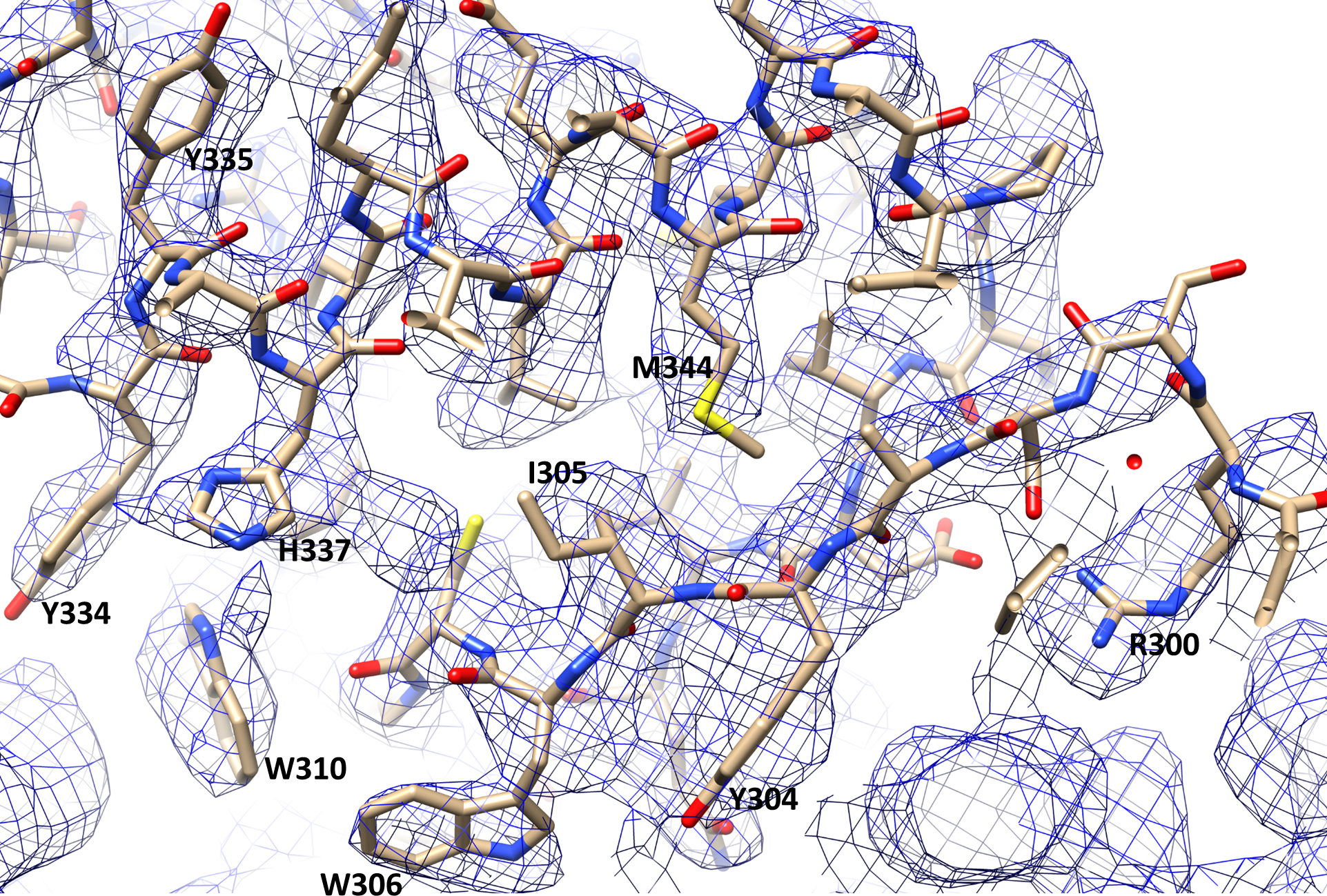

Supplement: Supplementary file 1 [file viruses-15-01517-s001.zip › Supplementary_Figure_S2.png]

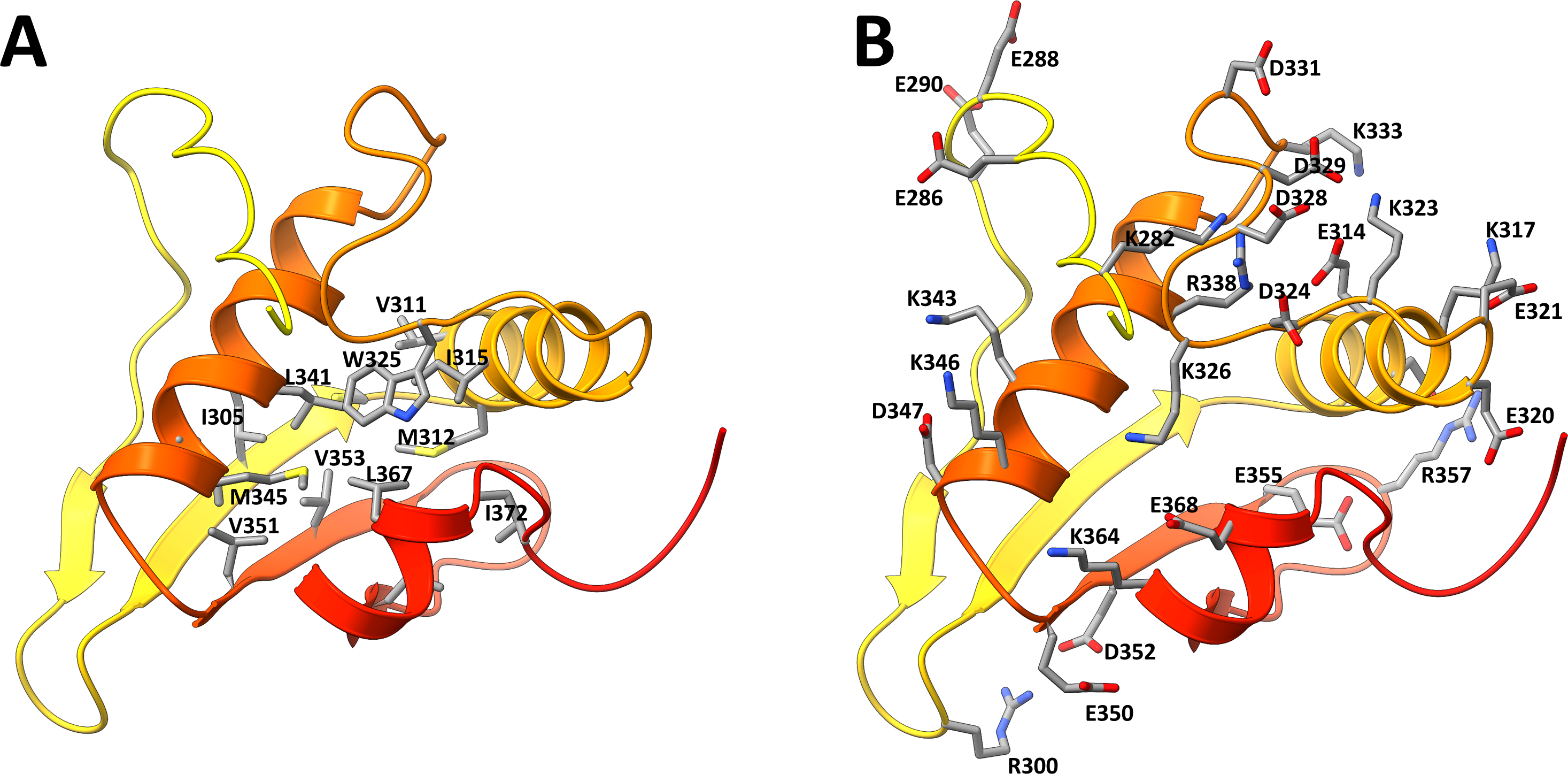

Supplement: Supplementary file 1 [file viruses-15-01517-s001.zip › Supplementary_Figure_S3.PNG]

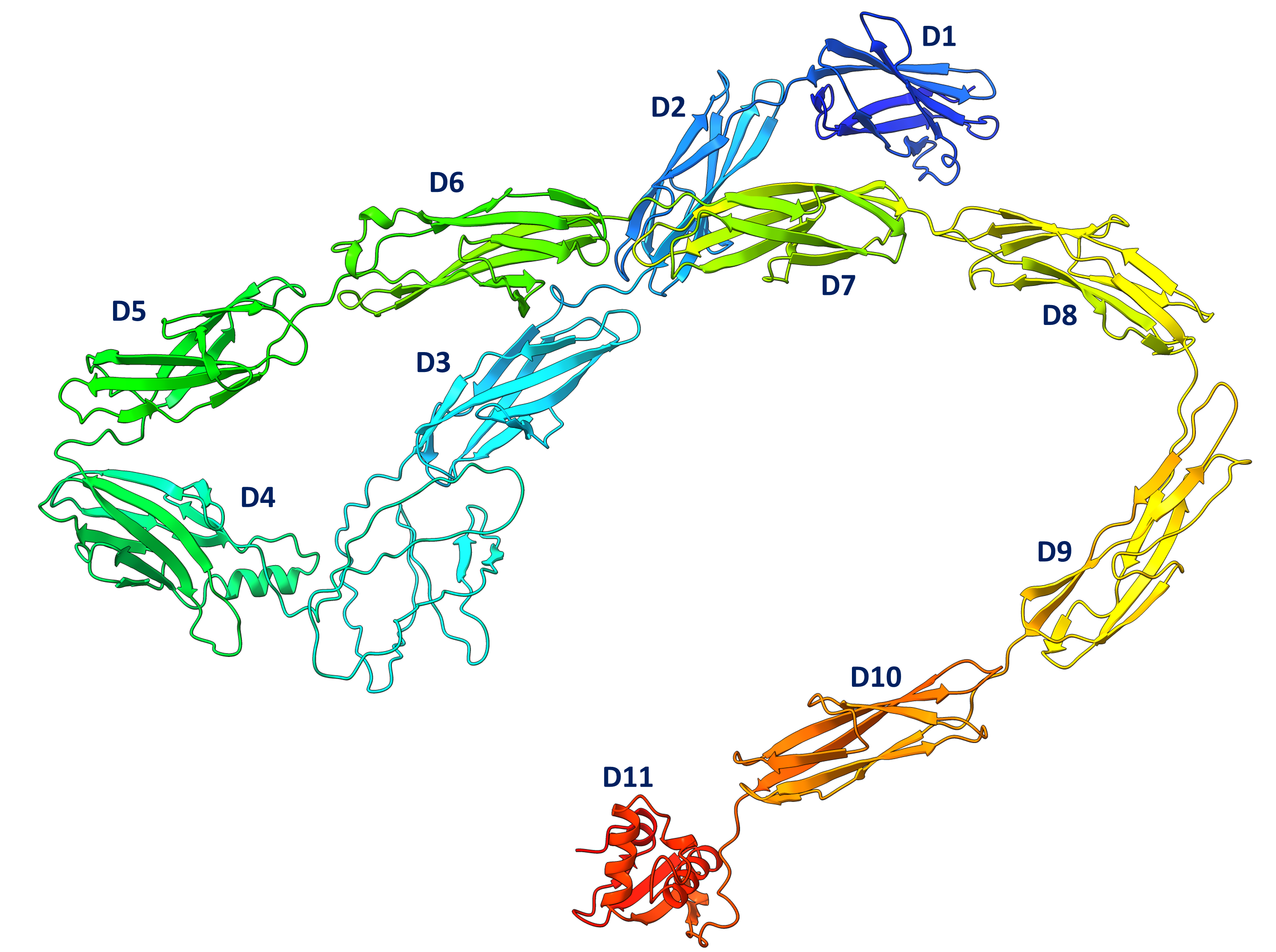

Supplement: Supplementary file 1 [file viruses-15-01517-s001.zip › Supplementary_Figure_S4.png]

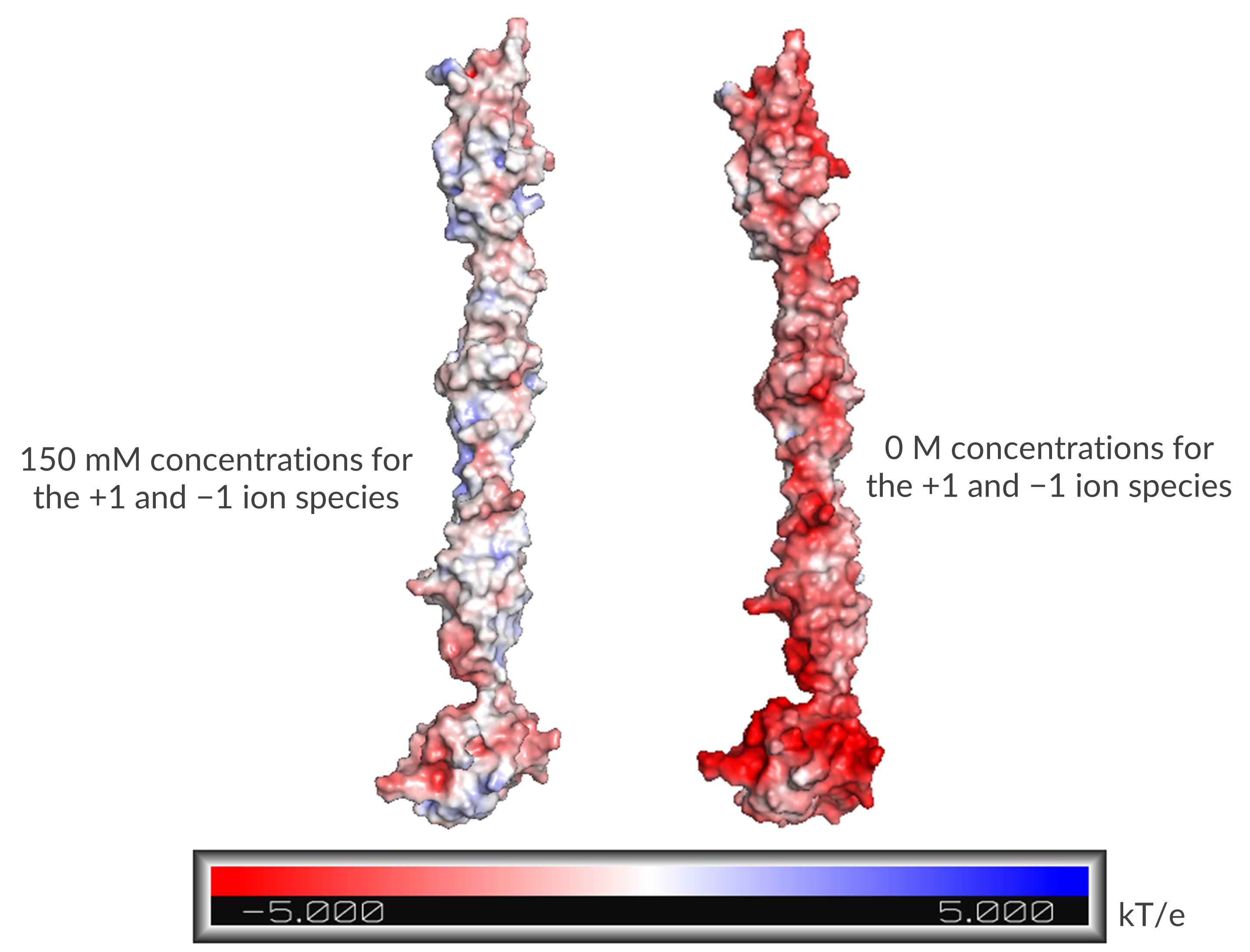

Supplement: Supplementary file 1 [file viruses-15-01517-s001.zip › Supplementary_Figure_S5.PNG]

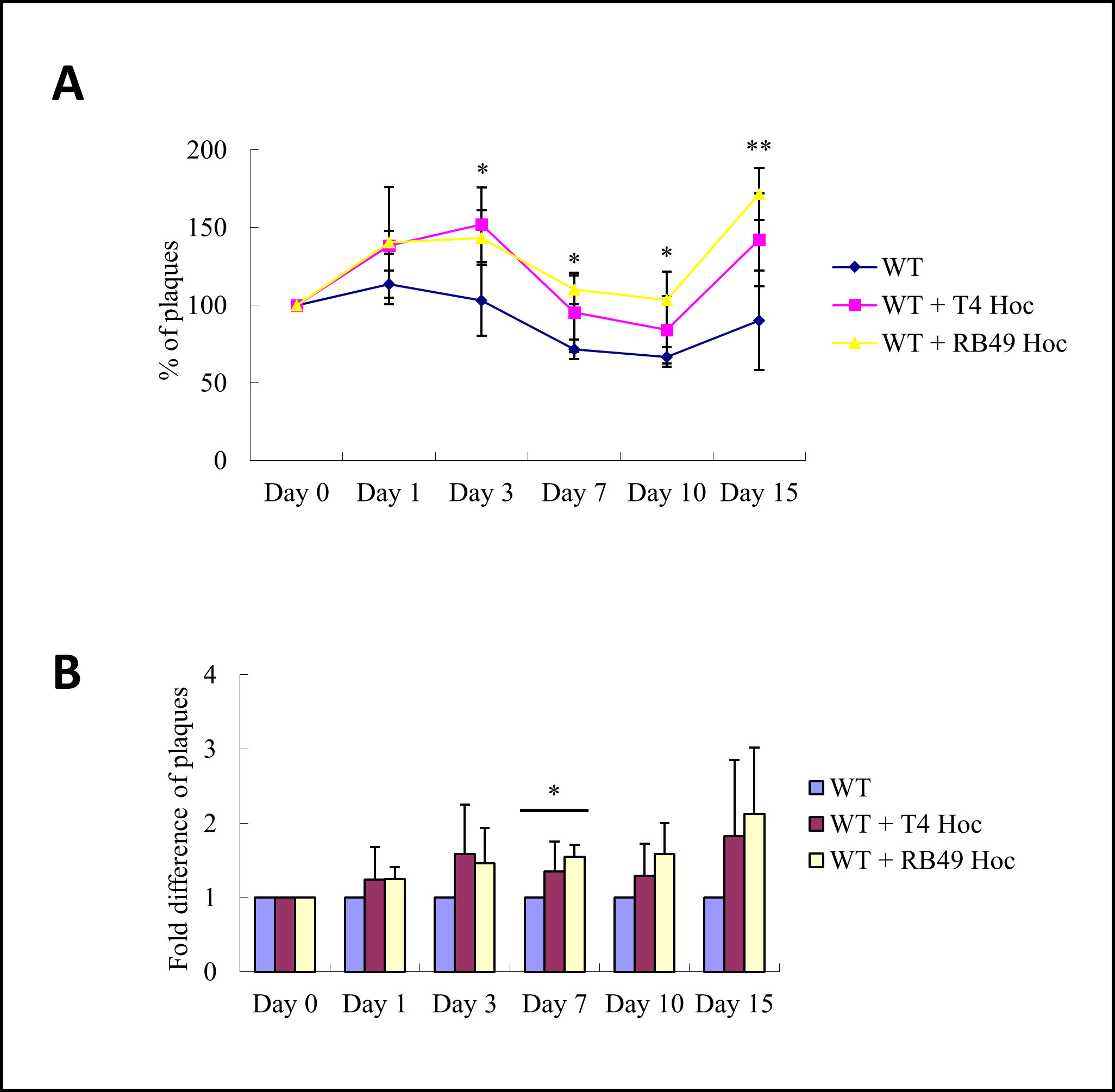

Supplement: Supplementary file 1 [file viruses-15-01517-s001.zip › Supplementary_Figure_S6.PNG]
