# Supplementary material for: Structure and Function of Hoc—A Novel Environment Sensing Device Encoded by T4 and Other Bacteriophages
Source: Viruses. 2023 Jul 7;15(7):1517. doi: 10.3390/v15071517 (PMC10385173; doi:10.3390/v15071517)
Supplement: Supplementary file 1 [file Supplementary_File_S1.pdf › Supplementary_File_S1.pdf]

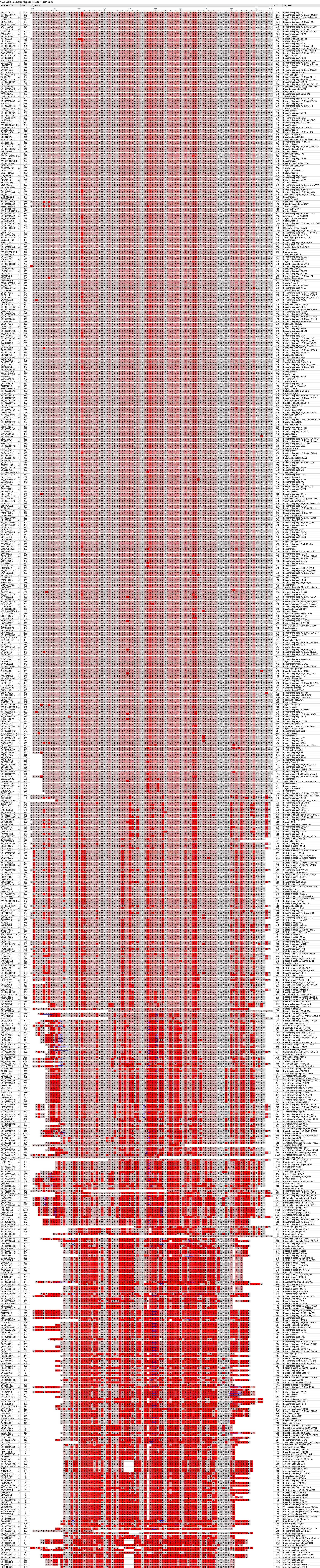
NCBI Multiple Sequence Alignment Viewer, Version 1.23.1
Sequence ID
Start
Alignment
End
Organism
